# Cariogenic Risk Modifies the Effect of Marginal Adaptation on Caries Adjacent to Posterior Composite Restorations: A Stratified Multivariate Analysis

**DOI:** 10.3390/clinpract16090163

**Published:** 2026-09-03

**Authors:** Andrei Georgescu, Sorin Andrian, Cristina-Angela Ghiorghe, Galina Pancu, Claudiu Topoliceanu, Ionuţ Tărăboanţă, Irina Nica, Răzvan Brânzan, Cristina Dascălu, Maria-Alexandra Mârţu, Mihaela Sălceanu

**Affiliations:** Faculty of Dental Medicine, Grigore T. Popa University of Medicine and Pharmacy Iasi, Universitatii Street 16, 700115 Iasi, Romania; andrei.georgescu@umfiasi.ro (A.G.); sorin.andrian@umfiasi.ro (S.A.); cristina.ghiorghe@umfiasi.ro (C.-A.G.); galina.pancu@umfiasi.ro (G.P.); ionut-taraboanta@umfiasi.ro (I.T.); nica.irina@umfiasi.ro (I.N.); razvan-constantin.branzan@umfiasi.ro (R.B.); cristina.dascalu@umfiasi.ro (C.D.); maria-alexandra.martu@umfiasi.ro (M.-A.M.); mihaela.salceanu@umfiasi.ro (M.S.)

**Keywords:** composite restoration, CARS, cariogenic risk, marginal adaptation

## Abstract

**Background/Objectives:** Caries adjacent to restorations or sealants (CARS) is among the most frequent biological causes of composite restoration failure, resulting from the interplay between the patient’s cariogenic risk and restoration-related factors such as marginal adaptation. The study aimed to investigate whether cariogenic risk modifies the effect of marginal adaptation on the occurrence of caries adjacent to posterior composite restorations (CARS), using risk-stratified univariate analysis and a clustering-adjusted approach, to identify associated clinical, socio-demographic, and local risk factors. **Methods:** This retrospective cross-sectional study evaluated 453 posterior direct composite resin restorations (Class I, Class II) in 88 patients at the Faculty of Dental Medicine, “Grigore T. Popa” University, Iași, Romania. CARS and marginal adaptation were assessed using FDI Criteria. Patients were classified as high or low cariogenic risk using a multifactorial institutional protocol. Univariate analysis and Generalized Estimating Equations (GEE) accounting for within-patient clustering were performed. **Results:** CARS prevalence was 27.9% in high cariogenic risk patients versus 3.15% in low cariogenic risk patients. Univariate analysis identified high cariogenic risk (OR = 11.904), deficient marginal adaptation (OR = 25.929–69.475), restoration age of 3–5 years (OR = 3.045), and Class II configuration (OR = 2.597) as significant risk factors. GEE analysis, adjusted for clustering of restorations within patients, confirmed high cariogenic risk (OR = 12.597) and deficient marginal adaptation (OR = 121.320) as independent correlates of CARS. A significant interaction between the two (*p* < 0.001) showed that marginal adaptation remained the dominant local risk factor in low cariogenic risk patients, while its association with CARS was largely attenuated in high-risk patients. **Conclusions:** GEE analysis confirmed high cariogenic risk and deficient marginal adaptation as independent correlates of CARS, with marginal adaptation acting as the dominant local risk factor only in low-risk patients.

## 1. Introduction

Caries adjacent to restorations or sealants (CARS) represents the most frequently reported biological reason for restoration repair or replacement decisions [1,2,3,4,5]. CARSs represent a complex and multifactorial process combining the etiological mechanisms of conventional caries with the specific characteristics of the restoration and restorative material involved [6,7]. When left undetected or untreated, CARS may progress to clinically significant pulpal complications [8], while in Class II restorations, the risk of periodontal complications arises from subgingival marginal discrepancies, proximal overhangs, and inadequate contact restoration, leading to plaque retention, gingival inflammation, and progressive attachment loss [9,10].

Three categories of factors impact the quality of direct composite restorations: patient-related factors, operator-related factors, and material-related factors [11]. A research group further clarified that the occurrence of CARS with composite resins is partially material-dependent, as composites have been associated with a higher incidence of caries compared to amalgam [12,13]. Risk factors significantly contributing to composite restoration failure by CARS were demographic factors, patient risk factors (high cariogenic risk, occlusal stress, smoking status, parafunctions, periodontal status, Class II restoration), dental group, cervical extension of restoration margins, and adhesive technique [14].

Marginal adaptation at the interface between a restoration and the cavity walls is recognized as both a clinician-dependent and a material-related risk factor for CARS, since the quality of this interface reflects operator skill, procedural choices, and the intrinsic properties of restorative materials. Clinician skill is considered the second most influential factor—after patient-related risk—affecting both restoration longevity and the incidence of CARS [15,16]. Operator experience and focus during restorative procedures directly influence long-term marginal integrity [17]. Any adhesive failure, nanometric porosities, or microleakage pathways within the hybrid layer can progressively widen under occlusal loading and hydrolytic degradation, ultimately allowing oral fluids and bacterial enzymes to penetrate the interface and initiate secondary carious lesions [18].

Comprehensive caries management must integrate biological, social, behavioral, cultural, systemic, and dental risk factors. Individual cariogenic risk is recognized as one of the most significant patient-related risk factors for the failure of direct composite resin restorations [19]. Cariogenic risk is one of the factors most strongly associated with restoration longevity, with high-risk patients presenting failure rates nearly three times higher than those recorded in low-risk patients [20,21]. The clinical relevance of cariogenic risk assessment prior to restorative treatment is underscored by evidence demonstrating that risk-stratified intervention can effectively reduce the cariogenic loading, both for primary dental caries and CARS [22,23]. Although cariogenic risk and marginal adaptation have each been independently associated with CARS, previous studies have generally analyzed them as parallel or additive predictors, without formally testing whether cariogenic risk modifies the clinical impact of marginal adaptation [6,8,12]. This interaction remains unexplored, limiting the translation of existing evidence into individualized, risk-stratified decisions regarding restoration monitoring, repair, or replacement.

Although cariogenic risk and marginal adaptation have each been independently associated with CARS [17,24], previous studies have generally treated risk factors for composite restoration failure as parallel, additive contributors [24], without formally testing whether cariogenic risk modifies the effect of marginal adaptation on CARS occurrence. This distinction is clinically relevant, as individualized, risk-stratified caries management is increasingly recommended [24]; however, no previous study has formally established whether cariogenic risk acts as an effect modifier of restoration-related local factors.

The study aimed to investigate whether cariogenic risk modifies the effect of marginal adaptation on the occurrence of caries adjacent to posterior composite restorations (CARS), using risk-stratified univariate analysis and a clustering-adjusted approach, to identify associated clinical, socio-demographic, and local risk factors.

We hypothesized that both high cariogenic risk and deficient marginal adaptation would be independently associated with an increased occurrence of CARS, and that cariogenic risk would significantly modify the effect of marginal adaptation on CARS occurrence.

The objectives of the study were:

1. To analyze the distribution of CARS in relation to age, cariogenic risk, and local restoration parameters;
2. To identify significant risk factors and odds ratios associated with CARS in posterior direct composite restorations stratified by cariogenic risk group (high vs. low);
3. To estimate how the probability of CARS occurrence varies across distinct clinical risk profiles and to determine whether the identified associations remain robust when accounting for the non-independence of restorations evaluated within the same patient.

## 2. Materials and Methods

### 2.1. Study Design

This retrospective cross-sectional study enrolled patients who underwent clinical and radiographic re-evaluation between January and March 2026. These patients were selected after applying the inclusion and exclusion criteria, from an initial pool of 312 patients eligible for posterior composite direct restorations between January 2021 and December 2024 at the Clinical Learning Base of the Faculty of Dental Medicine, “Grigore T. Popa” University of Medicine and Pharmacy, Iași, Romania.

A previous sample size calculation using G*Power software (version 3.1) for binary logistic regression indicated a minimum of 390 restorations were required to detect the effect of interest. Of the 312 patients initially screened, 162 were excluded: 125 for incomplete clinical records; 37 for posterior composite restorations in restoring cavities with ICDAS codes 03 (microcavitary) or 06 (large cavities). The remaining 150 eligible patients were subsequently randomly sampled to obtain the final study sample of 88 patients.

The group of selected patients had a mean age of 23.32 ± 3.449 years and comprised 48 males (mean age 22.96 ± 3.886 years) and 40 females (mean age 23.75 ± 2.826 years), with no statistically significant difference in age distribution between genders (*p* = 0.053).

Inclusion criteria were:

- occlusal or proximo-occlusal composite restorations;
- vital molar or premolar teeth;
- restoring cavities classified as ICDAS 04 and 05;
- restorations aged 1–2 years to 3–5 years at the time of evaluation;
- patients with documented cariogenic risk assessment.

Exclusion criteria were:

- incomplete clinical records;
- restoring cavities classified as ICDAS 03 (microcavitary) or 06 (extended).

All restorations were placed by sixth-year dental students under direct supervision of an experienced clinician. To minimize operator-related variability and ensure procedural consistency across all direct posterior composite restorations, a standardized clinical protocol was strictly followed at each stage of the restorative procedure: selective-stepwise caries excavation using electric handpieces at 800 rpm without water cooling, selective acid etching of enamel margins (37% phosphoric acid, 30 s), application of a two-step total-etch adhesive system (Adper Single Bond, 3M ESPE, Seefeld, Germany), and incremental oblique composite layering (Filtek Z250, 3M ESPE, Seefeld, Germany) with standardized light-curing intensity (≥1000 mW/cm^2^) and time (40 s each layer). Whenever a step of the procedure could not be completed by the student in strict accordance with the standardized protocol, the supervising clinician intervened directly and completed the step.

The research was carried out in accordance with the ethical principles of the Declaration of Helsinki and received approval from the Ethics Committee of “Grigore T. Popa” U.M.Ph. Iasi (Romania). All patients were informed about the study objectives and provided written informed consent.

### 2.2. Clinical Evaluation of CARS and Marginal Integrity

All restorations were evaluated between January 2026 and March 2026 by four examiners using the FDI World Dental Federation clinical criteria [25,26] for CARS and marginal adaptation. All four examiners were blinded to each other’s scores. Clinical examination included visual inspection, short air drying, and tactile assessment with a 250-μm probe. CARSs were diagnosed and classified using FDI Criterion 12 (Table 1) [25,26]. Marginal adaptation was assessed using FDI Criterion 6 (Table 2) [25,26]. A structured photographic and clinical calibration was performed among the four examiners prior to the study. All four examiners scored the same set of 30 standardized clinical photographs of posterior composite restorations in two sessions, with the second session two weeks after the first session, to minimize recall bias. Also, the four examiners evaluated 20 restorations in vivo, applying the same visual, air-drying, and tactile assessment protocol (250-μm probe) used throughout the study. The inter-examiner reliability was assessed by using the intraclass correlation coefficient (ICC). A two-way random-effects model was used to calculate ICC values, as recommended for ordinal multi-category rating scales assessed by more than two examiners [27]. The range of values for interpretation of ICC was as follows: <0.50, poor; 0.50–0.75, moderate; 0.75–0.90, good; and >0.90, excellent reliability [27]. The ICC values obtained for the four examiners were as follows: [0.84] (95% CI: [0.74–0.91]) for CARS (FDI Criterion 12) and [0.78] (95% CI: [0.66–0.97]) for marginal adaptation (FDI Criterion 6) in the photographic calibration, and [0.88] (95% CI: [0.79–0.94]) and [0.80] (95% CI: [0.66–0.90]), in the clinical calibration. These values indicate good inter-examiner agreement, a level of agreement consistent with previously reported inter-examiner reliability for FDI-based assessment of composite restorations [28].

For the purpose of binary logistic regression analysis, CARS outcome was dichotomized as follows: restorations scored FDI 12.1–12.3 were classified as clinically acceptable (success), while restorations scored 12.4–12.5 (requiring repair/replacement) were classified as clinically unacceptable (failure). Marginal adaptation was dichotomized at score 6.3 (excellent/very good/good/satisfactory: 6.1–6.3; deficient: 6.4–6.5).

### 2.3. Cariogenic Risk

Cariogenic risk was assessed using a structured clinical protocol developed within the Discipline of Cariology and Operative Dentistry, Faculty of Dental Medicine, Iași, Romania, following the multifactorial risk assessment paradigm established in the literature [29,30,31,32,33,34,35,36]. This paradigm defines cariogenic risk through the interplay of biological risk factors, behavioral risk factors, and disease indicators—domains reflected in the three components of our assessment protocol (anamnestic risk factors, intraoral risk factors, and cariogenic risk indicators).

The protocol comprised three domains of evaluation. The first domain covered anamnestic risk factors as follows: systemic diseases; unhealthy dietary habits; unsatisfactory oral hygiene; low socioeconomic status; low fluoride exposure. The second domain comprised intraoral risk factors as follows: caries experience (DMFT/DMFS scores > 10%); presence of active carious lesions; presence of dental sepsis, defined as pulpal exposure, ulceration, fistula, or abscess (PUFA index); biofilm retention factors (dental incongruences, orthodontic appliances, prosthetic restorations); and exposed root surfaces. The third domain assessed cariogenic risk indicators: presence of visible white-spot lesions on smooth surfaces; restorations placed within the previous 3 years; and dentinal caries detectable by radiography. Patients were classified as presenting high cariogenic risk if they fulfilled at least one of the following criteria: (a) the presence of at least two intraoral risk factors together with at least one cariogenic risk indicator; or (b) the presence of dental sepsis, considered a major risk factor independently sufficient for high-risk classification. Patients who did not meet any of these criteria were classified as presenting low cariogenic risk. The binary classification was adopted to enable stratified comparative analysis of the relation between cariogenic risk and CARS, consistent with current clinical cariogenic risk stratification [19,33].

### 2.4. Data Analysis

The independent variables evaluated as risk factors for CARS were as follows:

- Socio-demographic: gender (male vs. female).
- Local: Black’s cavity classification (Class I vs. Class II); restoration age (1–2 years vs. 3–5 years); arch location (maxilla vs. mandible); dental group (molar vs. premolar).
- Cariogenic risk (high vs. low).

The statistical analysis was conducted using SPSS version 31.0. Analyzed parameters were described using frequency distributions and descriptive statistics. Chi-squared tests were used to assess associations between categorical variables. Univariate analysis was performed to identify risk factors and odds ratios (OR with 95% CI) for CARS occurrence in the global sample and in each cariogenic risk subgroup. Given that the analyzed dataset contained multiple restorations per patient (453 restorations from 88 patients, mean value of 5.14 per patient), a binary logistic regression model was constructed using Generalized Estimating Equations (GEE), specifying patient identification number as the clustering variable for restorations. Covariates included the risk factors that will be identified through univariate analysis, together with the interaction terms between cariogenic risk and each of the other three factors. This approach addresses the violation of the independence assumption inherent to the data (restorations from the same patient are not independent) and allows us to assess whether high cariogenic risk, as a contextual variable, modifies the effect of the other factors on secondary caries occurrence.

## 3. Results

### 3.1. Descriptive Analysis: Posterior Composite Restorations

A total of 453 composite resin restorations were evaluated (Table 3).

The sample was slightly female-predominant (55.2% vs. 44.8% male) and comprised predominantly Class I cavities (79.2%) over Class II (20.8%), with a roughly even split between restorations aged 1–2 years and 3–5 years.

Restorations were similarly distributed between the maxilla and mandible and were markedly more frequent in molars than premolars (81.7% vs. 18.3%).

Most restorations were evaluated in patients with low cariogenic risk (70.0%) rather than high cariogenic risk (30.0%).

Marginal adaptation scores were concentrated at the lower end of the scale, with scores 6.1–6.2 together accounting for the large majority of restorations (82.8% combined), while deficient scores (6.3–6.5) comprised a small minority (17.2% combined).

CARS distribution was skewed toward absence of secondary caries (score 12.1, 68.9%), with clinically unacceptable scores (12.4–12.5) representing a minority of the sample (10.6% combined).

### 3.2. Relationship Between CARS and Marginal Adaptation

The relationship between marginal adaptation and CARS was statistically significant (Pearson Chi-squared: *p* < 0.001), with a moderate, directly proportional correlation between the two variables (Spearman ρ = 0.520, *p* < 0.001) (Table 4). Marginal adaptation and CARS were significantly related (Pearson Chi-squared, *p* < 0.001), with a moderate, directly proportional correlation (Spearman ρ = 0.520, *p* < 0.001) (Table 4). Restorations free of CARS (score 12.1) were almost always marginally acceptable (scores 6.1–6.2), while every restoration with the most severe CARS (score 12.5) showed deficient marginal adaptation (score 6.5)—and the categories in between followed the same pattern, with deficient margins becoming steadily more common as CARS severity increased. Intermediate CARS categories (scores 12.2–12.4) showed a corresponding gradient, with poor marginal adaptation scores increasingly frequent alongside more advanced CARS.

### 3.3. Univariate Analysis: CARS Risk Factors

Univariate analysis of the socio-demographic, clinical, and restoration variables in relation to success/failure (CARS 12.4–12.5) was performed in the high cariogenic risk group (Table 5) and the low cariogenic risk group (Table 6). In the high cariogenic risk subgroup, deficient marginal adaptation remained the only clearly significant risk factor (*p* < 0.001), with odds ratios of 40.286 (95% CI: 12.156–133.513) for score 6.4. Restoration age of 3–5 years reached borderline significance (OR = 2.375, 95% CI: 0.986–5.722, *p* = 0.05), while cavity class, gender, arch location, and dental group showed no statistically significant association with restoration status in this subgroup. In the low cariogenic risk subgroup, socio-demographic and local restoration variables did not reach statistical significance. Deficient marginal adaptation was the only factor significantly associated with CARS (*p* < 0.001); this association was stronger than in the high cariogenic risk group: OR = 50.500 (95% CI: 10.152–251.196) for score 6.4.

### 3.4. Generalized Estimating Equations (GEE): Risk Factors Interacting with CARS

In the GEE (Generalized Estimating Equations) model, we specified patient identification number as the clustering variable for restorations, and used as covariates the four risk factors identified through univariate analysis, namely cavity class, restoration age, cariogenic risk, and deficient marginal adaptation (≥6.4), to which we added the interaction terms between cariogenic risk and each of the other three factors. The constructed model—with a binomial distribution, logit link, an exchangeable working correlation matrix, and robust (sandwich) standard errors—was viable (QIC = 202.483; QICC = 205.293); the intra-patient correlation estimated by the working correlation matrix was negligible (−0.007); once patient clustering was correctly specified, restorations from the same patient were not more similar to one another than restorations from different patients. Within the GEE model, cavity class, restoration age, cariogenic risk, and marginal adaptation were entered as covariates, together with the interaction terms between cariogenic risk and each of the other three factors (Table 7). Class II (*p* = 0.481) and restoration age 3–5 years (*p* = 0.617) were not significantly associated with CARS. High cariogenic risk showed a significant, independent association with CARS (*p* = 0.005): patients with high cariogenic risk had 12.597 times higher odds of presenting with CARS than those with low risk. Marginal adaptation ≥ 6.4 was significantly and independently associated with CARS (*p* < 0.001): patients with deficient marginal adaptation had 121.320 times higher odds of presenting with CARS than those with adequate adaptation. The Class II × high cariogenic risk interaction was not significant (*p* = 0.429): Class II restorations were not associated with different CARS risk in high-risk patients when compared to low-risk patients. The restoration age 3–5 years × high cariogenic risk interaction was also not significant (*p* = 0.257), showing no differential association in high-risk patients. The marginal adaptation ≥ 6.34 × high cariogenic risk interaction was significant (*p* < 0.001): in patients with low cariogenic risk, deficient marginal adaptation was associated with a 121.320-fold increase in odds, but this dropped to 5.459 in patients with high cariogenic risk. The association between deficient marginal adaptation and CARS was largely masked in high-risk patients by the dominant effect of cariogenic risk itself.

### 3.5. Model-Estimated Probabilities of CARS by Clinical Profile

Based on the fitted GEE model equation (*p* = 1/(1 + e^−^_r_), where z = constant + B × variables), the model-estimated probabilities of CARS occurrence were calculated for 16 distinct clinical patient profiles, defined by cariogenic risk level, cavity class, restoration age, and marginal adaptation score (Table 8). These values are exploratory estimates derived from the fitted GEE model and should not be interpreted as clinically validated risk estimates, as the model has not yet been externally validated in an independent patient cohort.

The model-estimated probabilities of CARS occurrence across the 16 clinical profiles derived from the fitted GEE model ranged from 0.45% (low cariogenic risk, Class II cavity, restoration age 3–5 years, adequate marginal adaptation) to 52.22% (high cariogenic risk, Class II cavity, restoration age 3–5 years, deficient marginal adaptation). Deficient marginal adaptation was the factor most strongly associated with elevated CARS probability, though its impact varied by cariogenic risk profile: in low cariogenic risk patients, the shift from adequate to deficient marginal adaptation leads to a substantial increase in CARS probability (from 0.83% to 50.22%), whereas in high cariogenic risk patients, this same shift had a smaller relative effect (from 9.48% to 36.29%).

## 4. Discussion

One of the objectives of our study was to examine how frequently secondary caries adjacent to restorations (CARS) develops in patients with direct posterior composite resin restorations, and how this phenomenon distributes across different levels of cariogenic risk. Beyond establishing prevalence, we measured how strongly CARS occurrence relates to marginal adaptation quality and to a broader set of clinical and socio-demographic variables. Mapping these relationships allows clinicians to identify which risk factors are actionable and where targeted intervention is most likely to reduce restoration failure rates.

### 4.1. Cariogenic Risk Stratification and CARS Prevalence

Over the past 10–15 years, research has shifted toward identifying individual risk factors influencing composite restoration survival, with individual risk factors being reported as general variables in specific studies [34]. A joint ORCA/EFCD consensus review [29] concluded that while the restorative material itself has some influence on CARS, further factors such as the presence and size of restoration gaps, patients’ caries risk, and the dentist’s experience seem more relevant. This conclusion positions cariogenic risk as the primary modifiable determinant of CARS, independent of material choice. The patient and the operator are the two main factors impacting restoration longevity and risk of CARS, both being more relevant than the specific restorative protocol or material [26].

These findings justify the stratification of the study group by cariogenic risk level and support the hypothesis that high cariogenic risk constitutes a persistent biological substrate that composite restoration cannot neutralize in the absence of prior risk-targeted intervention. We classified patients according to cariogenic risk using an institutional protocol that shares the core conceptual structure of validated multifactorial systems such as CAMBRA and the ADA Caries Risk Assessment tool, evaluating disease indicators, biological risk factors, and anamnestic/behavioral factors [22,26,31,33]. One methodological choice in our protocol has a direct interpretive consequence: assigning independent sufficiency to dental sepsis (PUFA index) for high-risk classification may have shifted borderline patients into the high-risk group relative to systems such as the Cariogram or the Caries Risk Semaphore (CRS), and likely contributed to the markedly elevated odds ratio observed for cariogenic risk (OR = 12.60) compared with previous reports [35]. Because different cariogenic risk assessment tools have been shown to classify the same patients inconsistently [36,37], direct comparison of our cariogenic risk odds ratios with studies using CAMBRA-, Cariogram-, or ADA-based classifications should be made cautiously: differences in risk definitions, rather than true biological differences, may account for part of the variability in effect sizes across the literature.

In our study, CARS prevalence was 27.9% in the high cariogenic risk group, and only 3.15% in the low cariogenic risk group. Although cariogenic risk has been evaluated in numerous studies, only a few research groups included it as a covariate in multivariate regression models when predicting restoration survival [38,39]. In a systematic review and meta-analysis, direct composite restoration failure rates were 3.2% at 5 years and 4.6% at 10 years in high cariogenic risk patients, compared with only 1.2% and 1.6%, respectively, in low-risk patients [38]. A research group demonstrated a 4.4-fold increase in direct composite restoration failure risk associated with high cariogenic risk, CARS representing the most frequent cause of failure [39]. High cariogenic risk detected at re-evaluation in non-compliant patients can be considered a risk factor for CARS [40,41,42]. In a survey of Norwegian dentists, 52.1% of respondents ranked cariogenic risk management among the most important factors in preventing restorative failure due to CARS, which was reported as the most frequent cause of failure in Class II composite resin restorations [43]. Statistically significant differences in the clinical performance of composite resin restorations were found for low cariogenic risk patients when compared to the high cariogenic risk group [44]. While the restorative material exerts a moderate influence on CARS prevalence, cariogenic risk is the dominant patient-level risk factor [45].

### 4.2. Marginal Adaptation and Cariogenic Risk as Independent Correlates of CARS

Cavity class, restoration age, cariogenic risk, and marginal adaptation were the only variables carried forward into the GEE model, having been selected on the basis of univariate significance; gender, arch location, and dental group were excluded at this stage, as they showed no significant association with CARS in either cariogenic risk subgroup. This result is consistent with published data showing these factors are associated with higher CARS risk [40,41,42,43,44,45,46].

In the binary logistic regression model of the GEE (Generalized Estimating Equations) type, we specified patient identification number as the clustering variable for restorations, and used as covariates the four risk factors identified through univariate analysis, namely cavity class, restoration age, cariogenic risk, and deficient marginal adaptation (≥6.4), to which we added the interaction terms between cariogenic risk and each of the other three factors. These four covariates were retained because they had already been shown, in univariate analysis, to carry independent explanatory value for CARS occurrence; the GEE model was therefore not an exploratory re-analysis but a robustness check applied to the same set of established predictors, now under a clustering-adjusted framework. Because up to 15 restorations were evaluated per patient, treating these observations as independent would risk underestimating standard errors and overstating the precision of the associations identified. By modeling patient identity as the clustering variable, with an exchangeable working correlation structure and robust standard errors, the GEE model corrects for this while keeping the odds ratios interpretable. The negligible intra-patient correlation (−0.007) shows that restorations from the same patient did not share unmeasured characteristics beyond those already captured by the covariates. The associations identified were therefore not attributable to unmodeled intra-patient dependence. Once clustering was properly accounted for, the magnitude of association remained substantial—particularly for marginal adaptation. High cariogenic risk and deficient marginal adaptation remained the only variables significantly and independently associated with CARS, while cavity class and restoration age remained non-significant, consistent with the univariate analysis. The significant interaction between marginal adaptation and cariogenic risk was also preserved in the GEE model. The attenuation of the marginal adaptation effect in high-risk patients (from a 121.320-fold to a 5.459-fold increase in odds) indicates that once cariogenic risk is high, the biological environment predisposing to secondary caries becomes the dominant factor, largely overriding the contribution of local restoration quality. In low-risk patients, by contrast, the integrity of the marginal seal appears to be the main safeguard against secondary caries, since the systemic cariogenic burden is minimal. This supports a risk-stratified approach to restoration monitoring and recall scheduling, rather than uniform surveillance intervals across all patients regardless of cariogenic risk profile.

The effect of high cariogenic risk on CARS prevalence in our study was larger than those previously reported in the literature. Significantly higher OR for high cariogenic risk likely reflects a combination of sample-specific characteristics. The relatively young age of our patients and the short follow-up period of 1–5 years tend to amplify early biological effects before material degradation becomes the dominant failure mechanism. Also, the specific patient population of the university clinic setting is consistently reported to present with higher cariogenic risk profiles than those seen in private practice [2].

Deficient marginal adaptation (score ≥ 6.4) was the most strongly associated factor, with an OR of 121.320 (*p* < 0.001) in the low cariogenic risk subgroup. The significant interaction between poor marginal adaptation and high cariogenic risk (*p* < 0.001), with a decrease in OR from 121.320 to 5.459 in the high cariogenic subgroup, points to a ceiling effect: above a certain threshold of cariogenic burden, marginal quality loses much of its discriminative power, and the biological substrate drives CARS occurrence regardless of restoration marginal integrity. In patients with low cariogenic risk, by contrast, the mechanical integrity of the restoration becomes the decisive local determinant of CARS. This observation aligns with Askar et al. (2020), who concluded that cariogenic risk is a more relevant determinant factor of CARS than restoration gap size or material type [24]. The dual nature of CARS etiology—biological and mechanical—is thus preserved in our model: cariogenic risk dominates when high, while marginal adaptation becomes the primary determinant factor when cariogenic risk is low.

In the present study, deficient marginal adaptation showed the strongest individual association with CARS among patients with low cariogenic risk. This finding must be nuanced when analyzed in relation to the patient’s cariogenic risk profile. In patients with high cariogenic risk, the effect of deficient marginal adaptation is significantly attenuated, with cariogenic risk taking over as the dominant factor in the clinical picture—a biological ceiling effect. One possible explanation lies in the multifactorial nature of secondary caries, whose pathogenetic mechanisms are governed by the same risk factors as primary carious lesions. In patients with high cariogenic risk, the oral environment favors CARS occurrence even in the presence of adequate marginal adaptation. On the other hand, in patients with low cariogenic risk, deficient marginal adaptation represents the determining local factor in CARS occurrence. This differentiation underscores the importance of individual cariogenic risk assessment in interpreting the clinical significance of marginal defects and in optimizing therapeutic decisions (non-intervention vs. repair).

The marginal adaptation parameter in direct posterior composite resin restorations is determined by a constellation of clinician-controlled procedural variables; each variable is able to influence microleakage and CARS susceptibility. At the preparation stage, acid etching of enamel margins [47,48] and dentine [49] reduces microleakage risk, while selective caries excavation does not compromise marginal integrity [50]. Glass ionomer liners exceeding 1 mm increase failure risk [51,52], whereas thinner glass ionomer or calcium hydroxide liners reduce microleakage [53,54]. The open-sandwich technique additionally provides cariostatic activity and reduced polymerization contraction at cervical margins [55,56]. The restorative phase introduces further variables: adequate moisture control during placement is consistently associated with lower failure rates [57,58]; photopolymerization protocol affects pre-gel stress and degree of cure at the adhesive interface [59,60]; the choice of bonding technique and the adhesive system play a critical role in polymerization shrinkage stress, and determines the long-term marginal adaptation quality of composite restorations [61,62]; incremental oblique or centripetal layering mitigates polymerization stress in high C-factor cavities [62]; the use of flowable composites decrease cervical microleakage, but has no consistent advantage over resin-modified glass ionomer liners [63,64,65,66]; and improper finishing technique can stress an interface already weakened by polymerization shrinkage contraction, leading to marginal enamel microfractures [67,68]. Restorative technique is not an independent determinant of marginal adaptation and CARS when the biological substrate is properly managed [69,70].

The inclusion of marginal adaptation as a variable associated with CARS must be discussed. The co-occurrence of FDI scores 6.5 and 12.5 introduces a degree of overlap between predictor and outcome. However, this overlap does not invalidate the regression analysis. First, marginal adaptation was dichotomized into adequate (6.1–6.2) and deficient (6.3–6.5), using 6.3 as the threshold score. Scores 6.3 and 6.4 were observed in both success and failure restoration groups. Thus, marginal adaptation retains meaningful predictive value across the full range of clinical scores observed in this study. Second, the complete co-occurrence of scores 6.5 and 12.5 reflects a clinical reality. Advanced marginal breakdown and extensive CARS do not occur independently—both represent the final stage of the same restoration failure process.

### 4.3. Statistical Interaction and Biological Meaning

The interpretation of the statistical test results, and of the interaction between risk factors for CARS, cannot be dissociated from understanding the role played by the interaction terms within the structure of the GEE model. The introduction of an interaction term between cariogenic risk and marginal adaptation fundamentally alters the meaning of the main effect coefficients relative to the model without interaction. In the absence of interaction, the odds ratios (ORs) associated with marginal adaptation express the estimated average effect at a global level. Once the interaction term is included, the same odds ratio becomes specific to the low cariogenic risk group, while the OR specific to the interaction term itself expresses the magnitude of the difference in effect between restorations evaluated in low cariogenic risk patients and those evaluated in high cariogenic risk patients. In high cariogenic risk patients, the cariogenic oral environment influences the increased occurrence of CARS regardless of the quality of marginal adaptation. Under these conditions, cariogenic risk becomes a major risk factor. The data presented support the interpretation of this phenomenon as a biological ceiling effect of cariogenic risk. High cariogenic risk establishes a threshold of aggressiveness of the cariogenic oral environment above which the influence of deficient marginal adaptation progressively attenuates. This finding reinforces the dual—biological and mechanical—nature of CARS etiology, while simultaneously highlighting cariogenic risk control as the primary objective in the prevention strategy for secondary caries adjacent to composite resin restorations. Moreover, CARS could be interpreted in future studies considering the general oral environment regarding salivary biomarkers in caries-susceptible vs. caries-resistant individuals [71,72,73].

### 4.4. Limitations

As a retrospective cross-sectional study, the present design does not allow determination of the temporal sequence between marginal adaptation, cariogenic risk, and CARS occurrence, precluding causal inference; consequently, all reported relationships should be interpreted as statistical associations rather than causal or predictive effects.

A potential limitation of the present study relates to the academic clinical setting in which all restorations were placed. Sixth-year dental students, working under continuous faculty supervision, performed all restorative procedures. Although all restorations were placed under the direct supervision of university teaching assistants with a minimum of 20 years of clinical experience, following a standardized restorative protocol at every stage, the identity of the individual student-operator was not systematically recorded and could therefore not be accounted for as a covariate in the statistical analysis. While this may constrain the direct generalizability of the results to private dental settings, the supervised academic environment ensured a degree of procedural consistency, with all operators following a strictly standardized restorative protocol at every restorative stage. The homogeneity of the operator pool supports the reproducibility of the observed outcomes. The use of a university clinic patient population and supervised student operators is consistent with the design of published longitudinal studies evaluating the clinical performance of composite restorations [74,75].

A methodological limitation concerns the absence of standardized CARS diagnostic criteria across studies, as well as the lack of calibration between evaluator teams from different research groups; this can be a source of systematic bias that limits direct comparisons between published studies. The young and homogeneous study population drawn from a university dental clinic limits the generalizability of results to patient populations seen in private dental practice, where cariogenic risk profiles, restoration materials and techniques, as well as operator experience differ significantly. The relatively short restoration age of 1–5 years favors the detection of early biological failures driven by cariogenic risk, while potentially underrepresenting mechanical failure modes (bulk fracture, wear) that tend to emerge at longer follow-up intervals [76,77].

Although conceptually aligned with established multifactorial frameworks such as CAMBRA and the ADA Caries Risk Assessment tool, our institutional protocol was not directly compared against these validated instruments within the study sample. The GEE model and the probability estimates it produced were developed and tested on a single internal dataset, with no external validation in an independent patient cohort. This limits their immediate applicability as a clinical risk-stratification tool. Some odds ratios in the GEE model, particularly those involving marginal adaptation, carried wide confidence intervals, reflecting pronounced imbalance in event rates across subgroups rather than sparse data. All subgroup cell counts were adequate, but the magnitude of these associations should still be interpreted with caution; larger, multi-center studies with more balanced subgroup distributions are needed to refine these estimates.

### 4.5. Recommendations for Future Research

Future research should extend follow-up beyond five years and involve multiple centers, since the present retrospective design favored the detection of early biological failures over mechanical complications such as bulk fracture or wear, which tend to appear later in a restoration’s service life.

Since operator-related variability is a recognized determinant of restoration longevity and CARS risk, future studies should prospectively record operator-level identifiers to allow adjustment for potential operator-related clustering effects.

Researchers should extend studies to potential new materials, medications, and formulations used inside the oral cavity regarding performance, longevity, integration, and biocompatibility over time [78,79,80,81,82,83].

## 5. Conclusions

Univariate analysis confirmed that high cariogenic risk, deficient marginal adaptation, restoration age, and Class II cavities were all independently associated with increased CARS occurrence in the global sample. In high cariogenic risk patients, marginal adaptation remained significantly associated with CARS, while in low cariogenic risk patients, it was the major factor behind restoration failure related to CARS.

High cariogenic risk and deficient marginal adaptation were identified as independent correlates of CARS. In patients with high cariogenic risk, marginal adaptation exerted minimal influence on CARS occurrence; in those with low cariogenic risk, compromised marginal integrity was the primary determinant of secondary caries.

## Figures and Tables

**Table 1:** FDI Criterion 12—CARS [25,26].

Score	Rating	Description
12.1	Clinically excellent/very good	No caries/demineralization at the restoration margin detectable after air drying.
12.2	Clinically good	First visible signs of a non-cavitated caries lesion at the restoration margin detectable after air drying.
12.3	Clinically satisfactory	Established, non-cavitated caries lesion or microcavity at the restoration margin detectable without air drying.
12.4	Clinically unsatisfactory	Localized dentin cavity (width > 250 μm, depth > 2 mm) at the restoration margin. Repair is possible.
12.5	Clinically poor	Extensive dentin cavity at the restoration margin. Repair not possible/reasonable.

Criteria: visual examination, short air drying, and 250-μm probe. Scores 12.1–12.3 are clinically acceptable (sufficient); scores 12.4–12.5 are clinically unacceptable and require intervention.

**Table 2:** FDI Criterion 6—Marginal Adaptation [25,26].

Score	Rating	Description
6.1	Clinically excellent/very good	Ideal marginal adaptation of the restoration to the dental hard tissue after air drying. No marginal gap detectable by gentle probing.
6.2	Clinically good	Slight deficiencies of marginal adaptation after air drying. Minor, superficial marginal gap(s) or ditching.
6.3	Clinically satisfactory	Distinct deficiencies of marginal adaptation without air drying: marginal gap(s) (width < 250 μm and/or depth < 2 mm).
6.4	Clinically unsatisfactory	Localized but severe deficiencies of marginal adaptation: width ≥ 250 μm and/or depth ≥ 2 mm marginal gap(s). Partially loose/lost restoration. Repair is possible.
6.5	Clinically poor	Generalized and severely compromised marginal adaptation: width ≥ 250 μm and/or depth ≥ 2 mm. Complete loose/lost restoration. Repair not possible/reasonable.

Criteria: visual examination, short air drying, and 250-μm probe. Scores 6.1–6.3 are clinically acceptable (sufficient); scores 6.4–6.5 are clinically unacceptable and require repair or replacement.

**Table 3:** Descriptive analysis of posterior composite restorations and CARS Indices.

		*n*	%
Gender	male	203	44.8
	female	250	55.2
Class	class I	359	79.2
	class II	94	20.8
Age (filling)	1–2 ys	216	47.7
	3–5 ys	237	52.3
Location	MX	225	49.7
	MD	228	50.3
Location	M	370	81.7
	PM	83	18.3
Cariogenic risk	high	136	30.0
	low	317	70.0
Marginal adaptation	6.1	92	20.3
	6.2	283	62.5
	6.3	42	9.3
	6.4	16	3.5
	6.5	20	4.4
CARS	12.1	312	68.9
	12.2	43	9.5
	12.3	50	11.0
	12.4	32	7.1
	12.5	16	3.5
Total		453	100.0

**Table 4:** Distribution of CARS related to marginal adaptation FDI score.

		Secondary Caries										Total	
		12.1		12.2		12.3		12.4		12.5			
		*n*	%	*n*	%	*n*	%	*n*	%	*n*	%	*n*	%
Marginal Adaptation	6.1	90	28.8%	-	-	2	4.0%					92	20.3%
	6.2	202	64.7%	37	86.0%	32	64.0%	12	37.5%			283	62.5%
	6.3	14	4.5%	6	14.0%	14	28.0%	8	25.0%			42	9.3%
	6.4	6	1.9%	-	-	2	4.0%	8	25.0%			16	3.5%
	6.5	-	-	-	-			4	12.5%	16	100.0%	20	4.4%
Total		312	100.0%	43	100.0%	50	100.0%	32	100.0%	16	100.0%	453	100.0%

Pearson Chi-squared: *p* < 0.001: highly statistically significant; Spearman Correlation coefficient: rh = 0.520; mod-erate, directly proportional.

**Table 5:** Univariate analysis of the socio-demographic, clinical and restoration features according to the restoration status (success vs. failure)—high cariogenic risk.

		Secondary Caries—Evaluation				*p*-Value †	OR (95% CI)
		Failure		Success			
		*n*	%	*n*	%		
Gender	male	22	57.9%	48	49.0%	0.351	-
	female	16	42.1%	50	51.0%		
Class	class II	16	42.1%	28	28.6%	0.130	-
	class I	22	57.9%	70	71.4%		
Age (filling)	3–5 ys	30	78.9%	60	61.2%	0.05 *	2.375 (0.986 ÷ 5.722)
	1–2 ys	8	21.1%	38	38.8%		
Location	MX	16	42.1%	58	59.2%	0.073	-
	MD	22	57.9%	40	40.8%		
Location	M	34	89.5%	74	75.5%	0.071	-
	PM	4	10.5%	24	24.5%		
Marginal adaptation						<0.001 **	
	6.1	-		12	12.2%		
	6.2	10	26.3%	56	57.1%	0.020 *	-
	6.3	4	10.5%	26	26.5%	<0.001 **	6.347 (2.739 ÷ 14.704)
	6.4	6	15.8%	4	4.1%	<0.001 **	40.286 (12.156 ÷ 133.513)
	6.5	18	47.4%	-		<0.001 **	-
Total		38	100.0%	98	100.0%		

† Chi-squared test; * *p* < 0.05 statistically significant; ** *p* < 0.01 statistically highly significant.

**Table 6:** Univariate analysis of the socio-demographic, clinical and restoration features according to the restoration status (success vs. failure)—low cariogenic risk.

		Secondary Caries—Evaluation				*p*-Value †	OR (95% CI)
		Failure		Success			
		*n*	%	*n*	%		
Gender	male	4	40.0%	129	42.0%	1.000	-
	female	6	60.0%	178	58.0%		
Class	class II	2	20.0%	48	15.6%	0.661	-
	class I	8	80.0%	259	84.4%		
Age (filling)	3–5 ys	6	60.0%	141	45.9%	0.523	-
	1–2 ys	4	40.0%	166	54.1%		
Location	MX	6	60.0%	145	47.2%	0.527	-
	MD	4	40.0%	162	52.8%		
Location	M	8	80.0%	254	82.7%	0.686	-
	PM	2	20.0%	53	17.3%		
Marginal adaptation						<0.001 **	
	6.1	-		80	26.1%		
	6.2	2	20.0%	215	70.0%	0.071	-
	6.3	4	40.0%	8	2.6%	<0.001 **	98.333 (18.819 ÷ 513.814)
	6.4	2	20.0%	4	1.3%	<0.001 **	50.500 (10.152 ÷ 251.196)
	6.5	2	20.0%	-	-	<0.001 **	-
Total		10	100.0%	307	100.0%		

† Chi-squared test; ** *p* < 0.01 statistically highly significant.

**Table 7:** GEE multivariate logistic regression results.

Parameter	B Coef.	Robust SE	*p*-Value	OR (95% CI)
Class II	−0.615	0.871	0.481	0.541 (0.098 ÷ 2.984)
Age (filling) 3–5 ys	−0.362	0.725	0.617	0.696 (0.168 ÷ 2.884)
High Cariogenic Risk	2.533	0.904	0.005 **	12.597 (2.142 ÷ 74.076)
Marginal adaptation ≥ 6.4	4.798	0.806	<0.001 **	121.320 (24.981 ÷ 589.190)
Class II × High Cariogenic Risk	0.754	0.953	0.429	2.126 (0.329 ÷ 13.750)
Age (filling) 3–5 ys × High Cariogenic Risk	0.875	0.771	0.257	2.398 (0.529 ÷ 10.867)
Marginal adaptation ≥ 6.4 × High Cariogenic Risk	−3.105	0.914	<0.001 **	0.045 (0.007 ÷ 0.269)
Constant	−4.789	0.857	<0.001 **	0.008 (0.002 ÷ 0.045)

*p* < 0.05 statistically significant; ** *p* < 0.01 statistically highly significant.

**Table 8:** CARS probability according to cariogenic risk and restoration parameters.

Cariogenic Risk	Cavity Class	Restoration Age	Marginal Adaptation	Probability of CARS
Low	I	1–2 years	<6.4	0.83% (reference)
Low	II	1–2 years	<6.4	0.45%
Low	I	3–5 years	<6.4	0.58%
Low	II	3–5 years	<6.4	0.31%
Low	I	1–2 years	≥6.4	50.22%
Low	II	1–2 years	≥6.4	35.3%
Low	I	3–5 years	≥6.4	41.27%
Low	II	3–5 years	≥6.4	27.53%
High	I	1–2 years	<6.4	9.48%
High	II	1–2 years	<6.4	10.75%
High	I	3–5 years	<6.4	14.89%
High	II	3–5 years	<6.4	16.74%
High	I	1–2 years	≥6.4	36.29%
High	II	1–2 years	≥6.4	39.56%
High	I	3–5 years	≥6.4	48.75%
High	II	3–5 years	≥6.4	52.22%

Interaction term: The interaction term Cariogenic Risk × Marginal Adaptation is included in the model equation only when both variables are simultaneously equal to 1 (i.e., high cariogenic risk and deficient marginal adaptation). Marginal adaptation: Score ≥ 6.4 denotes deficient marginal adaptation; score < 6.3 denotes adequate marginal adaptation.

## Data Availability

The original contributions presented in this study are included in the article. Further inquiries can be directed to the corresponding author.

## Abbreviations

The following abbreviations are used in this manuscript:

ADA	American Dental Association
AUC	Area Under the Curve
CAMBRA	Caries Management by Risk Assessment
CARS	Caries Adjacent to Restorations or Sealants
CEJ	Cemento-Enamel Junction
CI	Confidence Interval
CRS	Caries Risk Semaphore
DMFS	Decayed, Missing, Filled Surfaces
DMFT	Decayed, Missing, Filled Teeth
FDI	Fédération Dentaire Internationale (World Dental Federation)
ICCMS	International Caries Classification and Management System
ICDAS	International Caries Detection and Assessment System
MD	Mandible
MX	Maxilla
OR	Odds Ratio
ORCA	Organization for Caries Research
PM	Premolar
PUFA	Pulp involvement, Ulceration, Fistula, Abscess
SPSS	Statistical Package for the Social Sciences
